# Spinal metastases at the thoracolumbar junction – Influencing factors for surgical decision-making according to a multicentric registry

**DOI:** 10.1016/j.bas.2025.104198

**Published:** 2025-01-31

**Authors:** Vanessa Hubertus, Arthur Wagner, Arian Karbe, Leon-Gordian Leonhardt, Beate Kunze, Susanne Borchert, Fatma Kilinc, Michelle Mariño, Nitzan Nissimov, Charlotte Buhre, Marcus Czabanka, Marc Dreimann, Sven O. Eicker, Lennart Viezens, Hanno S. Meyer, Peter Vajkoczy, Bernhard Meyer, Julia S. Onken

**Affiliations:** a Department of Neurosurgery, Charité – Universitätsmedizin Berlin, corporate member of Freie Universität Berlin, Humboldt-Universität zu Berlin, and Berlin Institute of Health, Berlin, Germany; b Department of Neurosurgery, School of Medicine, Klinikum Rechts der Isar, Technical University of Munich, Munich, Germany; c Department of Neurosurgery, University Medical Center Hamburg-Eppendorf, Hamburg, Germany; d Division of Spine Surgery, Department of Trauma and Orthopedic Surgery, University Medical Center Hamburg-Eppendorf, Hamburg, Germany; e Spine Surgery, Orthopedic Hospital Markgröningen gGmbH, Markgröningen, Germany; f Department of Neurosurgery, University Hospital Frankfurt / Main, Frankfurt, Germany; g Department of Neurosurgery, Helios Hospital Berlin Buch, Berlin, Germany; h Department of Spine Surgery, Lubinus Clinicum Kiel, Germany; i German Consortium for Translational Cancer Research, DKTK, Part of the German Cancer Research Centre, Berlin, Germany

**Keywords:** Spinal metastasis, Spinal oncology, Thoracolumbar junction, Spine instrumentation, surgical indication

## Abstract

**Introduction:**

Spinal metastases at the thoracolumbar junction (TLJ) pose a significant risk for spinal instability and necessitate special considerations regarding surgical management. Longer patient survival due to improved oncologic therapies may justify extensive instrumented surgery.

**Research question:**

The aim of this study was to analyze the standard of care in a large multicentric cohort of patients with TLJ metastases regarding surgical decision-making, management, and associated morbidity.

**Material and methods:**

Patients with surgically treated spinal metastases at the TLJ between 2010 and 2022 were enrolled at five academic tertiary spine centers. Epidemiological, surgical, clinical, and outcome data was assessed retrospectively. Surgical management was sorted according to three groups: decompression (***i***), decompression and posterior instrumentation (***ii***), and decompression and 360° instrumentation with vertebral body replacement (***iii***). Sole biopsies or kypho-/vertebroplasties were excluded.

**Results:**

The inclusion criteria was met by 396 patients, of which 59 (15%) were treated with decompression (***i***), 235 (59%) with posterior instrumentation (***ii***), and 102 (26%) with additional vertebral body replacement (***iii***). The main factor for selection towards instrumentation was a higher SINS score (SINS 9 in ***ii***, 10 in ***iii*** vs. 7 in ***i***, p < 0.0001). Surgical complications occurred in 55 cases (14%), slightly more frequent following instrumentations (15% ***ii, iii*** vs. 8% ***i***, p = 0.427). Reoperations were necessary in 65 cases (16%), mostly due to SSI (n = 19, 29%), local recurrence (n = 15, 23%), and hardware failure (HWF) during follow-up (n = 9, 18%, ***i-iii***, p = 0.7853). HWF occurred significantly more frequent in cases with multisegmental metastases at the TLJ (p = 0.0002) which were treated with longer constructs (p = 0.0092). Median postoperative survival was 10 months. The occurrence of complications reduced postoperative survival drastically in all groups (p = 0.0023).

**Discussion and conclusion:**

In this large multicentric patient cohort with TLJ metastases, the majority of patients (85%) were treated with instrumented spine surgery. The main factor for patient selection towards instrumented surgery was a higher SINS score. Long instrumentations for multisegmental disease at the TLJ were identified with higher risk for hardware-failure during follow-up. In those patients, frequent follow-up imaging is warranted. As postoperative survival is drastically reduced by the occurrence of postoperative complications, it is imperative to carefully select the individually appropriate extent of surgery in order to avoid postoperative complications.

## Highlights

• Presentation of a large multicentric patient cohort with TLJ metastases, mostly (85%) treated with instrumented spine surgery.
• Instrumentations for multisegmental disease were identified with higher risk for hardware-failure during follow-up. Frequent follow-up imaging is warranted.
• Postoperative survival is drastically reduced by the occurrence of postoperative complications. Careful selection towards the individually appropriate extent of surgery is imperative in order to avoid postoperative complications.

## 1 Introduction

Spinal metastases occur in more than 50% of cancer patients, mostly in the thoracic and lumbar spine (Wagner et al., 2021; Onken et al., 2019). Especially metastases of the thoracolumbar junction (T11-L1) pose a significant risk for spinal instability and neurological deficits and necessitate special considerations regarding surgical advance (Wagner et al., 2021; Fehlings et al., 2009; Hubertus et al., 2021; Fisher et al., 2010). However, surgical management in this anatomic region is complex, holding a significant risk for related complications (Polly et al., 2009; Scheufler et al., 2011a).

Due to the evolution of systemic oncological therapies like immunotherapy and targeted therapies, survival of stage four cancer of most primaries is significantly increased, as is consequently the incidence of symptomatic bone metastases requiring treatment (Nater et al., 2018; Park et al., 2016; Dea et al., 2014; Barzilai et al.; Barzilai et al., 2018a; Ahmed et al., 2018; Luksanapruksa et al., 2017; Tokuhashi et al., 2014; Polly et al., 2009; Choi et al., 2015; Hu et al., 2022). The question remains how to consider improved prognosis and prolonged survival in these heterogenic and mostly palliative patients regarding the appropriate complexity of surgical therapy (Wagner et al., 2021; Luksanapruksa et al., 2017; Tokuhashi et al., 2014; Hirabayashi et al., 2003; Amelot et al., 2017; Choi et al., 2015).

In the treatment of spinal metastases, the *NOMS decision framework* by Laufer et al. is an established tool for guiding patient-centered and individualized therapy decisions (Laufer et al., 2013). Using this approach, individual risk and benefit for treatment is assessed by considering the patient's neurological and oncological status, specifications of the primary tumor like sensitivity to radio- and chemotherapy, mechanical considerations like spinal stability as assessed by the SINS score (Versteeg et al., 2016; Dosani et al., 2018; Fisher et al., 2010), and systemic factors like metastatic status and clinical performance (Barzilai et al., 2018a; Laufer et al., 2013; Bilsky et al., 2009). Established prognostic scoring models for spinal metastases are however not taking into consideration the recent advance of life-prolonging systemic oncological therapies (Tokuhashi et al., 2014; Amelot et al., 2017; Choi et al., 2013).

Especially for complex localizations with a high risk for spinal instability and neurological deficits like the junctional regions (cervicothoracic, thoracolumbar spine), no guidelines exist for choosing the accurate surgical strategy and decision-making is mostly based on a case-to-case basis (Choi et al., 2013; Tomita et al., 2001; Fehlings et al., 2009; Hubertus et al., 2021; Polly et al., 2009). Data on the durability of instrumentations or surgical revision rates during follow-up in these patients is scarce.

With this study we analyze one of the hitherto largest multicentric patient cohorts with surgical treatment of spinal metastases at the thoracolumbar junction (T11-L1) regarding factors influencing surgical decision-making, the choice and complexity of surgical management, and associated morbidity.

## 2 Material and methods

**Data collection:** Electronic patient records were collected at five participating tertiary neurosurgical and orthopedic spine centers throughout Germany. Records were retrospectively screened, anonymized and transferred towards the primary study center for analysis.

Inclusion criteria was set as surgically treated spinal metastases at the thoracolumbar junction (TLJ, spine segments T11, T12, and/or L1) at participating centers between 2010 and 2022, and patient age ≥18 years. Surgical approach was sorted according to three groups: ***i*** decompression only, ***ii*** decompression and posterior instrumentation, and ***iii*** decompression with 360° instrumentation and vertebral body replacement. In ***iii*** 360° vertebral column resections were included. Sole biopsies or kypho-/vertebroplasties without instrumentation and/or decompression were excluded.

As primary outcome parameter, the occurrence of postoperative surgical complications was set. Secondary outcome parameters included the occurrence of serious medical complications, length of hospital stay, postoperative neurological status and McCormick score, surgical revision rate, the occurrence of kyphosis and hardware-failure during follow-up, and postoperative patient survival.

Collected surgical data included details on the surgical technique, date of surgery, number of instrumented vertebrae, minimally-invasive or open approaches, use of neuronavigation, and the time of surgery from cut to suture.

To assess the patient's preoperative clinical status, following parameters were collected: Age at hospital admission, sex, date of primary diagnosis, primary tumor entity, status of systemic disease, mechanical pain due to spinal metastases, neurological deficits, acuteness of clinical presentation, and the following scores: McCormick, American Association of Anesthesology (ASA), and Karnofsky Performance Status (KPS).

Routine pre- and postoperative imaging (CT, MRI and/or X-ray) were retrospectively collected, and after anonymization sent to the primary study center for analysis. According to these images, the preoperative SINS of every metastatic vertebra at the TLJ (T11-L1) was assessed retrospectively.

**Data management and statistical analysis:** Data management was performed using REDCap® database and Microsoft Excel. Data analysis was performed using Graphpad Prism (version 9) and SPSS (IBM SPSS Statistics 29). Figures were created using Graphpad Prism (version 9), Sankeymatic.com and Biorender.com. Statistical significance was tested via one-way ANOVA with Bonferroni's post-hoc analysis or Kruskal-Wallis-test with Dunn's post-hoc analysis for multiple comparisons, depending on Shapiro Wilk test for Gaussian distribution. Categorial variables were evaluated for statistical significance via Chi-square testing. Survival was tested using Kaplan Meier analysis with Log-rank-test. Significance level was set at *p<0.05*. All statistical analyses were of exploratory nature.

## 3 Ethical approval

All procedures performed were in accordance with the ethical standards of the institutional research committee and with the 1964 Helsinki declaration and its later amendments or comparable ethical standards. Ethical approval (EA4/063/20) was granted by the institutional ethics board of the participating centers. Informed patient consent was waived according to the ethical approval, due to the collection of clinical data during standard treatment and the retrospective nature of the study.

## 4 Results

**Fig. 1:**
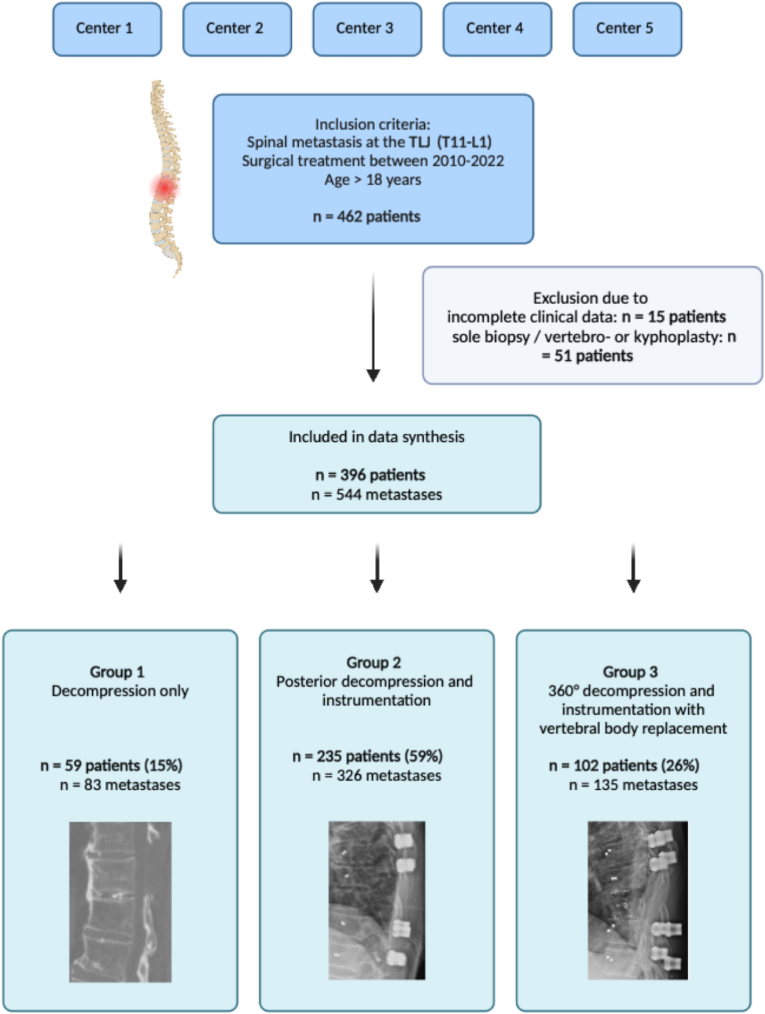
Overview of study recruitment and surgical groups.

**Study cohort:** A total of 462 patients suffering from spinal metastases at the TLJ (T11 – L1) were identified at five participating tertiary spine centers. Of those, 51 were excluded due to not meeting the inclusion criteria and 15 due to incomplete data availability, leaving 396 patients for data synthesis and analysis. Those patients suffered from a total of 544 spinal metastases at the TLJ. Treated with decompression without instrumentation (***i***) were 59 patients (15%, 83 metastases). Decompression and posterior instrumentation (***ii***) was performed in 235 cases (59%, 326 metastases), whilst 102 patients (26%, 135 metastases) were treated with decompression, 360° instrumentation and vertebral body replacement (***iii***). Group ***iii*** also included five cases of 360° vertebral column resections (*Overview of the study recruitment:* Fig. 1).

**Fig. 2:**
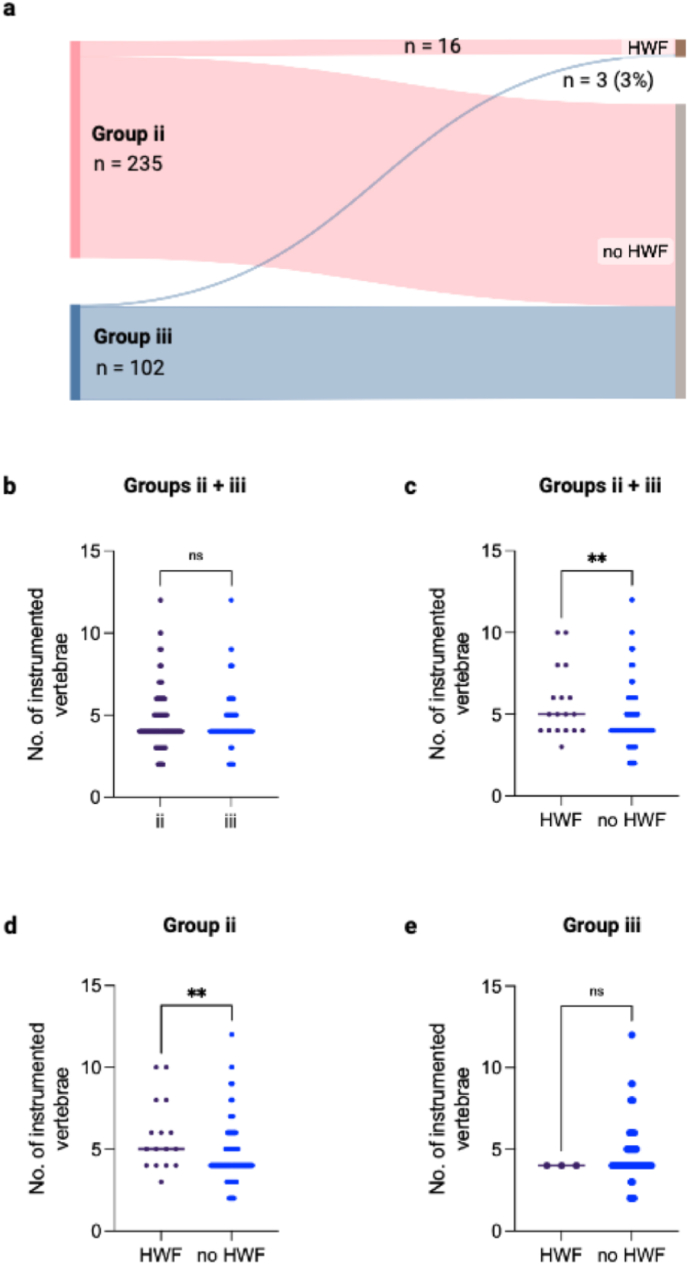
Data on the occurrence of hardware-failure during clinical follow-up in the instrumented groups (ii, iii), and relation to the length of instrumentations. a Occurrence of Hardware-failure during clinical follow-up in groups ii (n = 16) and iii (n = 3), b-e length of instrumentation in the different instrumented groups (b) and relation to the occurrence of hardware-failure during clinical follow-up in groups ii and iii (c), group ii (d), and group iii (e). Statistics: Student's *t*-test. Abbreviations: HWF – Hardware-failure. Statistics: Student's *t*-test. Abbreviation: HWF – Hardware failure.

Regarding the NOMS data (neurological, oncological, mechanical and systemic, by Laufer et al. (2013), Table 1, Suppl. 1), patients treated with decompression without instrumentation (***i***) presented more frequently with neurological deficits (64% in ***i*** vs. 34% and 28% in ***ii*** and ***iii***, p < 0.0001) and with worse initial McCormick score (p < 0.0001) than patients which received instrumentations (***ii*** and ***iii***). Clinical presentation in ***i*** was also more frequently acute (less than 48h since symptom onset, 38% ***i*** vs. 17% ***ii,*** 11% ***iii***, p = 0.0016). Patients treated with instrumentation presented with significantly higher rates of mechanical pain (73% and 74% in ***ii*** and ***iii*** vs. 53% in ***i***, p = 0.0046) and significantly higher SINS (9 in ***ii***, 10 in ***iii*** vs. 7 in ***i***, p < 0.0001).

**Table 1:** Demographic and NOMS (Neurologic, oncologic, mechanical and systemic, by Laufer et al.) data of the study cohort and surgical groups.

	All	*i*	*ii*	*iii*	p value
Patients, n (%)	396	59 (15)	235 (59)	102 (26)	n/a
Mean age (range)	65 (20–93)	64 (20–89)	66 (30–93)	63 (31–88)	0.19
Sex, n (%)	m 228 (58) f 168 (42)	m 38 (64) f 21 (36)	m 143 (61) f 92 (39)	m 47 (46) f 55 (54)	**0.02**
Neurological deficits, n (%)	146 (37)	38 (64)	79 (34)	29 (28)	**<0.0001** (i vs. ii, i vs. iii)
McCormick, n (%), *n=total values available*	1 - 224 (57) 2–71 (18) 3–37 (9) 4–39 (10) 5–21 (5), *n=392*	1 - 18 (31) 2–11 (19) 3–5 (9) 4–11 (19) 5–13 (22), *n=58*	1 - 140 (60) 2–37 (16) 3–27 (12) 4–20 (9) 5–8 (3), *n=232*	1 - 66 (65) 2–23 (23) 3–5 (5) 4–8 (8) 5 - 0 (0), *n=102*	**<0.0001**
Acuteness of presentation, n (%), *n=total values available*	Acute 60 (19) Subacute 60 (19) Nonacute 195 (62), *n=315*	Acute 20 (38) Subacute 10 (19) Nonacute 22 (42), *n=52*	Acute 30 (17) Subacute 32 (18) Nonacute 113 (65), *n=175*	Acute 10 (11) Subacute 18 (20) Nonacute 60 (68), *n=88*	**0.0016**
Pain (n, %)	279 (70)	31 (53)	174 (74)	74 (73)	**0.0046** (i vs. ii, i vs. iii)
Median SINS TLJ (range)	9 (3–18)	7 (3–16)	9 (3–18)	10 (3–17)	**<0.0001** (i vs. ii, i vs. iii)
>1 TLJ metastasis (n, %)	148	24 (29)	91 (28)	33 (24)	0.459
Metastatic segments whole spine (median, range)	4 (1–30)	5 (1–30)	5 (1–30)	3 (1–25)	**0.002** (i vs. iii, ii vs. iii)
Metastases other than spine (n, %)	236 (60)	34 (58)	151 (64)	51 (50)	0.05
Median KPS (range)	70 (20–100)	70 (20–100)	70 (20–100)	80 (30–100)	**0.0005** (i vs. iii, ii vs. iii)

Statistics: One-way ANOVA with Bonferroni post-hoc test for multiple comparisons, xi^2^ test for categorial data. Abbreviations: f – female, KPS – Karnofsky Performance Status, m – male, n/a – not applicable, SINS – Spinal instability neoplastic score, TLJ – Thoracolumbar junction.

**Surgical data:** Surgery with decompression and instrumentation with or without vertebral body replacement (***ii*** *and* ***iii***) took significantly longer than decompression alone (mean 142 min ***i*** vs. 196 min ***ii*** and 210 min ***iii***, p < 0.0001). In 82 cases (80%) with 360° instrumentation and vertebral body replacement (***iii***), a two-staged surgical approach was undertaken due to higher surgical complexity. In both groups ***ii*** and ***iii***, on average (mean) five vertebrae were instrumented (range 2–12). Intraoperative neuronavigation was utilized in 115 cases (49%) in group ***ii***, and in 60 cases (59%) in group ***iii***, with increasing use over the study period. About half of all navigated instrumentations in groups ***ii*** and ***iii*** were also conducted in a minimally-invasive (MIS) fashion (***ii*** 66 vs. ***iii*** 34 cases). In group ***i*** four cases (6%) received vertebro-/kyphoplasty (VPK) in addition to decompression and were therefore conducted with the use of neuronavigation in a MIS fashion (*Detailed overview of surgical data:* Table 2, Suppl. Fig. 3).

**Table 2:** Surgical data of the study cohort and surgical groups.

	All	*i*	*ii*	*iii*	p value
Total no. of surgeries (%)	396 (100)	59 (15)	235 (59)	102 (26)	n/a
Mean duration of surgery in min (range)	192 (40–762)	142 (40–378)	196 (61–762)	210 (82–626)	**<0.0001** (i vs. ii, i vs. iii)
More-staged surgeries, n (%)	90 (23)	0 (0)	8 (3)	82 (80)	n/a
Mean no. of instrumented vertebrae (range)	4 (0–12)	0 (0)	5 (2–12)	5 (2–12)	n/a
Navigation, n (%)	179 (45)	4 (6) *+ VPK*	115 (49)	60 (59)	n/a
MIS, n (%)	105 (27)	4 (6) *+ VPK*	66 (28)	34 (33)	n/a

Statistics: One-way ANOVA with Bonferroni post-hoc test for multiple comparisons. Abbreviations: MIS – minimally-invasive surgery, n/a – not applicable, VPK – Vertebro-/Kyphoplasty.

**Clinical outcome:** Following surgery, neurological status remained mostly stable (314 patients, 80%), while 70 patients (18%) showed neurological improvement and nine patients (2%) deteriorated. There were no between-group differences (p = 0.15) regarding neurologic outcome after surgery. Of all 396 patients, 52 (13%) suffered from serious medical complications following surgery, like deep venous thrombosis, pulmonary embolism, cardiac arrhythmia, or infections, independent of surgical group (7–16%, p = 0.136). Surgical complications occurred in 55 cases (14%), slightly more frequently after instrumentation (***ii*** and ***iii*** 15%, **i** 8%, p = 0.427). Most surgical complications were surgical site infections (SSI, n = 28, 51%), followed by hematoma (n = 11, 20%) and primary hardware malplacement (n = 5, 9%, ***ii*** and ***iii***). Type of surgical complications differed between groups (p < 0.0001), with the highest rate of SSI in ***ii*** (n = 23, 66%).

Reoperations were necessary in 76 cases (19%) and mostly due to SSI (n = 26, 33%), local tumor recurrence (n = 15, 19%) and hardware-failure during follow-up (n = 19, 24%). Mean time from initial spine surgery to reoperation was 12 months (range 0–90). Local tumor recurrence was a frequent reason for reoperations in all groups (***i*** n = 3, 5%, ***ii*** n = 8, 3%, ***iii*** n = 4, 4%). Primary hardware malplacement necessitating revision surgery occurred in three cases (1%) (***ii****)*.

Hardware-failure (HWF) in the form of screw loosening occurred in a total of 19 instrumented cases (5% of all instrumentations) during a mean clinical follow-up of 12 months. Of those patients, three were instrumented with vertebral body replacement (***iii***), and 16 without (***ii***). Regarding the length of instrumentations in patients with and withour HWF, no significant differences were found (median 5, range 2–12, both groups). However in both groups, HWF occurred more frequently in longer constructs (HWF: mean 6, no HWF: mean 5 instrumented vertebrae, p = 0.0092). Additionally, the number of metastastic vertebrae at the TLJ did differ significantly between cases with and without HWF (HWF: 8 patients (42%) with >1 metastasis at the TLJ, no HWF: 86 cases (27%), p = 0,0002). Other factors like SINS, the total number of spinal metastases including other spine regions or the occurrence of other surgical complications, did not differ. Kyphotic deformity during follow-up occurred only in four patients, and only one of them was associated with the occurrence of HWF (***ii***). (*Clinical outcome data:* Table 3, *Data on instrumented cases with and without hardware-failure:* Table 4.

**Table 3:** Clinical outcome data of the study cohort and surgical groups.

	All	*i*	*ii*	*iii*	p value
Total no. of patients (%)	396 (100)	59 (15)	235 (59)	102 (26)	n/a
Postoperative neurology, n (%)	Stable 314 (80) Ameliorated 70 (18) Deteriorated 9 (2)	Stable 19 (33) Ameliorated 15 (26) Deteriorated 1 (2)	Stable 188 (80) Ameliorated 38 (16) Deteriorated 8 (3)	Stable 84 (83) Ameliorated 17 (17) Deteriorated 0 (0)	0.15
Direct postop. medical complications, n (%)	52 (13)	4 (7)	37 (16)	11 (11)	0.136
Direct postop. surgical complications, n (%)	55 (14) - SSI 28 (51) - Hematoma 11 (20) - HWM 5 (9) - Others 11 (20)	5 (8) - SSI 1 (20) - Hematoma 3 (60) - Others 1 (20)	35 (15) - SSI 23 (66) - Hematoma 2 (6) - HWM 3 (9) - Others 7 (20)	15 (15) - SSI 4 (27) - Hematoma 6 (40) - HWM 2 (13) - Others 3 (20)	0.427 details **0.0059**
Reoperations due to complications, HWF or local recurrence, n (%)	76 (19) - SSI 26 (33) - Hematoma 10 (13) - HWM 3 (4) - HWF 19 (24) -Recurrence 15 (19) - Others 6 (6)	8 (12) - SS 1 (13) - Hematoma 3 (38) - Recurrence 3 (38) - Others 1 (13)	48 (20) - SSI 14 (29) - Hematoma 2 (4) - HWM 3 (6) - HWF 16 (33) - Recurrence 8 (17) - Others 5 (10)	17 (17) - SSI 11 (48) - Hematoma 5 (22) - HWF 3 (13) - Recurrence 4 (17) - Others 0 (0)	0.7853 details **<0.0001**
Mean time to reoperation in months (range)	12 (0–90)	21 (0–66)	7 (0–58)	18 (0–90)	0.0947
Mean hospital stay in days (range)	16 (2–68)	13 (2–39)	16 (3–68)	20 (4–66)	**<0.0001** (i vs. iii, ii vs. iii)
In-hospital mortality, n (%)	16 (4)	5 (8)	8 (3)	3 (3)	0.169
Median postop. follow-up in months (range)	11 (0–125)	12 (0–125)	10 (0–76)	15 (0–105)	0.065
Median postop. survival in months (range)	10 (0–68)	6 (0–39)	21 (0–68)	37 (0–59)	**0.0058**

Statistics: One-way ANOVA with Bonferroni post-hoc test or Kruskal-Wallis-test with Dunn's post-hoch test for multiple comparisons. Xi^2^ test for categorial variables. Survival tested by Kaplan Meier analysis with log-rang test. Abbreviations: HWM – Hardware malplacement, HWF – Hardware failure, ICU – intensive care unit, SSI – surgical site infection.

**Table 4:** Comparison of cases with and without hardware-failure during follow-up. Hardware-failure occurred in 19 of 337 instrumented cases (5%) after a mean of 12 months.

	Hardware-failure	No hardware-failure	p value
Patients, n (n groups)	19 (16 ***ii***, 3 ***iii***)	318 (219 ***ii***, 99 ***iii***)	n/a
Median SINS (range)	9 (1–16)	9 (1–18)	0.6274
>1 TLJ metastasis, n (%)	8 (42)	86 (27)	**0.0002**
Mean no. of metastatic segments whole spine (range)	5 (1–15)	4 (1–30)	0.5624
Mean no. of instrumented vertebrae (range)	6 (3–10)	5 (2–12)	**0.0092**
Kyphotic deformity, n (%)	1 (5)	3 (1)	0.2080
Other surgical complications, n (%)	SSI 5 (28) Hematoma 0 (0) HWM 1 (5)	SSI 20 (34) Hematoma 7 (12) HWM 2 (3)	0.3421

Statistics: Student's *t*-test for numeric variables, Xi^2^ test for categorial variables. Abbreviations: HWM – Hardware malplacement, SINS – Spinal Instability Neoplastic Score, SSI – surgical site infection, TLJ – Thoracolumbar junction.

**Fig. 3:**
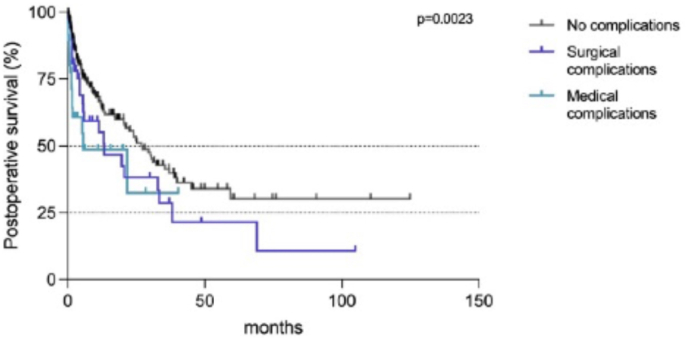
Postoperative survival (%) in months depending on the occurrence of postoperative complications (p = 0.0023). Statistics: Kaplan Meier survival analysis with log-rank test for between-group differences.

Mean hospital stay was 16 days (range 2–68) and differed between groups (***i*** 13, ***ii*** 16, ***iii*** 20, p < 0.0001) (see Fig. 2). In-hospital mortality was low with 16 cases in total (4% all groups, 3% ***ii*** and ***iii***, 8% ***i***, p = 0.169). On average (median), patients survived postoperatively for 10 months (range 0–68). Regarding the surgical group, the shortest postoperative survival was found in group ***i*** with a median of 6 months (vs. 21 months ***ii***, 37 months ***iii***, p = 0.0058). A distinct factor influencing postoperative survival was the occurrence of postoperative complications. While patients without any postoperative complications survived 27 months (median), survival was drastically reduced to 13 months with the occurrence of surgical complications, and even to 6 months with the occurrence of postoperative serious medical complications (p = 0.0023) *(Data on postoperative patient survival:* Fig. 3).

## 5 Discussion

This study presents of one of the largest multicentric cohorts of surgically treated patients with spinal metastases at the thoracolumbar junction. In the following, we discuss the clinical factors influencing surgical decision-making, management and associated morbidity.

In this cohort, surgical decision-making was influenced by the patient's *NOMS* criteria (neurological, oncological, mechanical and systemic) (Laufer et al., 2013). Of highest importance for patient-selection towards surgical instrumentation in this univariate analysis was the SINS. Metastatic lesions treated with instrumentations presented with higher SINS (***iii*** > ***ii***), while decompression alone was mostly performed in stable lesions. Moreover, patients receiving decompression alone (***i***) presented more frequently as neurological emergencies, and systemic tumor burden was highest in those patients.

As the junctional spine regions play a special role in the mechanical stability of the spine, it is important to especially consider spinal stability in the surgical treatment of junctional metastasis (Fehlings et al., 2009; Hubertus et al., 2021; Polly et al., 2009; Scheufler et al., 2011a). The SINS score as the most accepted score to assess the mechanical stability of spinal metastases already grants highest scores towards junctional location (3 of 3 points) (Fisher et al., 2010). While choosing the right treatment for (potentially) unstable junctional metastases, differences in biomechanical capacity of different instrumentations must also be considered, including the choice and the length of instrumentation itself, as well as additional fusion techniques and implant materials (Barzilai et al.; Versteeg et al., 2016; Dosani et al., 2018; Fisher et al., 2010). By using technological progress and the recent advances in radiosurgery, therapy concepts like minimally-invasive instrumentations and separation surgeries, procedure-related complications might be further reduced, enhancing adjuvant therapy planning and ameliorating postoperative patient outcome (Wagner et al., 2021; Barzilai et al.; Barzilai et al., 2018a; Zuckerman et al., 2016; Hubertus et al., 2022; Barzilai et al., 2018b). Although the optimal length of instrumentation alone remains under debate, the instrumentation of 5 vertebrae was the most frequently chosen strategy for treating monosegmental disease. Through the routine utilization of neuronavigation, longer instrumentations should pose no additional surgical risk. However in this cohort, longer instrumentations were frequently used in cases with multisegmental disease at the TLJ, and were thus associated with higher rates of postoperative HWF.

Compared to the broad literature on spinal metastases in general, the overall complication rate in this study was 14% and comparably low (Wagner et al., 2021; Barzilai et al., 2018a). As expected, postoperative complications consisted mostly of surgical site infections and hematomas. Not surprisingly, the rate of surgical complications differed between groups according to surgical complexity. Of all patients, 13% suffered from serious medical complications following surgery, like deep venous thrombosis, pulmonary embolism, cardiac arrhythmia, or infections, not depending on the surgical complexity. The occurrence of both surgical and medical postoperative complications was highest following instrumented spine surgery. Over the study period between 2010 and 2022, technological advances like navigated instrumentations and intraoperative 3D image-guidance were established at the participating centers, methods that are known to increase patient safety due to enhanced instrumentation accuracy, also prompting lower reoperation rates (Wagner et al., 2021; Hubertus et al., 2022; Hecht et al., 2018; Kendlbacher et al., 2022; Hussain et al., 2020; Härtl et al., 2013; Habib et al., 2021; Gelalis et al., 2012; Scheufler et al., 2011b).

HWF in the form of screw loosening occurred in this cohort in 19 instrumented cases (5% of all instrumentations) after a mean of 12 months. Of those patients, 16 were treated with posterior instrumentation, and three with additional vertebral body replacement. Only four patients presented with progressive kyphosis during follow-up. Factors identified with an associated higher statistical risk for HWF in this exploratory analysis were the existence of more than one spinal metastasis at the TLJ and a more extensive instrumentation length in these cases.

Multiple factors influenced postoperative patient survival in this study, like the surgical group, the underlying primary tumor, or the occurrence of postoperative complications. It is to be noted, that additional data on adjuvant treatment is not given, which will influence the clinical course during follow-up. However, the factor which influenced postoperative survival most in this exploratory analysis was the occurrence of postoperative complications. Thus, the occurrence of surgical complications reduced survival drastically from 27 to 13 months, and even to 6 months with the occurrence of serious medical complications. The reduction of postoperative survival due to the occurrence of postoperative complications is a well-known problem in the literature, and the most frequent surgical complication is wound infection (Park et al., 2016; Luksanapruksa et al., 2017; Sugita et al., 2016; Atkinson et al., 2016; Kumar et al., 2015; Sebaaly et al., 2018; Hu et al., 2022). Therefore, is is of utmost importance to reduce postoperative complications to a minimum, and to avoid infections with preventive measurements (Zimlichman et al., 2013; Magill et al., 2014; Voss and Hopman, 2014; Margaryan et al., 2020; Koch et al., 2023).

## 6 Conclusions

In this large multicentric patient cohort with metastases at the thoracolumbar junction, most patients (85%) were treated with instrumented spine surgery. The main factor for patient selection towards instrumented surgery was a higher SINS score. Long instrumentations for multisegmental disease at the TLJ were identified at risk for hardware-failure during follow-up. In those patients, frequent imaging follow-up is warranted. With the demonstrated patient selection for different surgical complexities, the rate of surgical complications was moderate with 14%. As postoperative survival is drastically reduced by the occurrence of postoperative complications, utmost care must be provided to select patients adequately for the individually appropriate extent of surgery and to avoid postoperative complications.

## Funding and acknowledgements

No special funding has been received for this article. VH is supported by the BIH Charité Clinician Scientist program. JSO is a BIH Charité clinical fellow.

## Author contributions

JO, VH, and PV designed the study concept. VH, AW and MM contributed the majority of cases. JO, VH, NN and CB performed data analysis and participated in data acquisition. VH and JO wrote the manuscript. AW, MM, AK, LGL, BK, SB, FK and CB contributed cases and participated in data acquisition. MC, MD, SOE, LV, HM, BM and PV revised the manuscript and contributed to data analysis.

## Availability of data and materials

The datasets supporting the conclusions of this article are included within the article and its additional file. Additional data can be provided by the corresponding author upon reasonable request.

## Ethical approval

All procedures performed in studies involving human participants were in accordance with the ethical standards of the institutional research committee and with the 1964 Helsinki declaration and its later amendments or comparable ethical standards. Ethical approval (EA4/063/20) was granted by the institutional ethics board of the Charité Ethics Committee and local ethics committees of participating centers.

## Declaration of competing interest

The authors declare that they have no known competing financial interests or personal relationships that could have appeared to influence the work reported in this paper.

## Abbreviations

ANOVA – Analysis of variance, ASA – American Association of Anesthesiology, CT – Computed Tomography, HWF – Hardware failure, HWM – Hardware malplacement, ICU – Intensive care unit, KPS – Karnofsky Performance Status, L – Lumbar vertebra, MIS – Minimally-invasive surgery, MRI – Magnetic Resonance Imaging, N/A – Not applicable, NOMS – Neurological, oncological, mechanical and systemic decision framework, SINS – Spinal Instability Neoplastic Score, SSI – Surgical site infection, T – Thoracic vertebra, TLJ – Thoracolumbar junction, VPK – Vertebro-/Kyphoplasty, Xi^2^ – Chi-square test.
