# Supplementary material for: Spinal metastases at the thoracolumbar junction – Influencing factors for surgical decision-making according to a multicentric registry
Source: Brain Spine. 2025 Jan 31;5:104198. doi: 10.1016/j.bas.2025.104198 (PMC11876765; doi:10.1016/j.bas.2025.104198)
Supplement: Multimedia component 1 [file mmc1.docx]

**Supplemental material with legends**

**Suppl. Fig.1:** Distribution of primary tumors in the different surgical groups (**a** = group 1, **b** = group 2, **c** = group 3), displaying a significantly different distribution of primary tumors between groups (p=0.0009).

**Suppl. Fig.2:** Postoperative survival (%) in months in the different surgical groups (p=0.0058).

**Suppl. Fig.3: a** Overview of the number of surgeries according to surgical groups from 2010-2022. **b** Increased amount of navigated spine instrumentationsand c increased amount of MIS instrumentations in **ii** and **iii**. **d** Surgical complication rate remaining similar in instrumented groups **ii** and **iii** between 2010-2022 (0-33%). Abbreviations: MIS – Minimally-invasive surgery.
